# Real-time, label-free monitoring of cell viability based on cell adhesion measurements with an atomic force microscope

**DOI:** 10.1186/s12951-017-0256-7

**Published:** 2017-03-22

**Authors:** Fang Yang, René Riedel, Pablo del Pino, Beatriz Pelaz, Alaa Hassan Said, Mahmoud Soliman, Shashank R. Pinnapireddy, Neus Feliu, Wolfgang J. Parak, Udo Bakowsky, Norbert Hampp

**Affiliations:** 1 Department of Chemistry, University of Marburg, Marburg, Germany; 2 Department of Physics, University of Marburg, Marburg, Germany; 3 Department of Pharmacy, University of Marburg, Marburg, Germany; 4 CIC biomaGUNE, San Sebastián, Spain; 5 Material Science Center, University of Marburg, Marburg, Germany

**Keywords:** AFM, Cell adhesion, Fast-screening measurement, Gold nanoparticles, Cytotoxicity, Cell viability

## Abstract

**Background:**

The adhesion of cells to an oscillating cantilever sensitively influences the oscillation amplitude at a given frequency. Even early stages of cytotoxicity cause a change in the viscosity of the cell membrane and morphology, both affecting their adhesion to the cantilever. We present a generally applicable method for real-time, label free monitoring and fast-screening technique to assess early stages of cytotoxicity recorded in terms of loss of cell adhesion.

**Results:**

We present data taken from gold nanoparticles of different sizes and surface coatings as well as some reference substances like ethanol, cadmium chloride, and staurosporine. Measurements were recorded with two different cell lines, HeLa and MCF7 cells. The results obtained from gold nanoparticles confirm earlier findings and attest the easiness and effectiveness of the method.

**Conclusions:**

The reported method allows to easily adapt virtually every AFM to screen and assess toxicity of compounds in terms of cell adhesion with little modifications as long as a flow cell is available. The sensitivity of the method is good enough indicating that even single cell analysis seems possible.

**Electronic supplementary material:**

The online version of this article (doi:10.1186/s12951-017-0256-7) contains supplementary material, which is available to authorized users.

## Background

The cell membrane is more than just a passive lipid bilayer barrier. Of special relevance, cell membrane proteins are an integral part of the cellular machinery concerning sensing and reacting to what surrounds the cell, through different processes such as signaling, transport and immune response. In particular, cell adhesion molecules and their main function, i.e., cell adhesion, are of prime importance on cell biology and medicine, being a key player on several biological processes such as tumor invasion and metastasis [1], stem-cell fate [2] and cell death and/or growth arrest [3]. Cell detachment, or loss of anchorage in adhesive cells, is a common marker of cell death [4], which could be monitored as a sign of cytotoxicity. For instance, intracellular signals caused by the intracellular accumulation of exogenic agents (e.g. toxins, drugs, nanoparticles, etc.) at toxic concentrations can in general cause cell detachment [5], followed by cell death.

In order to evaluate the safety of a new agent, variety of in vitro cell-based assays is often employed. One feasible strategy to evaluate the potential toxic effects of an unknown compound will be, in the first stage, to evaluate basal cytotoxicity (by using for instance screening assays), and second assess the specific types of toxicity [6] (i.e. to understand the cause cell injury). There are several cell-based assays used to evaluate cytotoxicity, including methods to monitor the function of organelles, cell viability, to track cellular components, etc. Cell viability assays are among the most frequently used methods in all form of cell cultures [7]. There are a variety of cell viability assays that could be used to monitor enzymatic activities or general metabolism, some of those assay include the resazurin and tetrazolium reduction, as well as protease activity methods [8].

Most frequently employed standard cytotoxicity methods to assess cell death, including cell viability and proliferation assays, rely on extrinsic labeling or reporter agents which, once internalized, interact with specific cell components providing a signal, typically colorimetric, fluorescent, or bioluminescent. The measured signal can be then related to different cellular parameters that are evaluated and associated in terms of cell viability, such as the activity of mitochondrial enzymes, for instance the succinate dehydrogenase, the intactness of cell membranes, adenosine triphosphate production, etc. [9]. The major limitation of these in vitro methods to evaluate cytotoxicity is that they may be affected by interferences between the compounds and the read-out signal. As example, metallic nanoparticles (NPs) may interact specifically or non-specifically with the reagent or substrate of the assay [10, 11]. Fluorescent NPs may cause crosstalk with fluorescence read-out of the assay. Further-more, some of the conventional toxicity methodologies are single endpoint assays, i.e., fail to provide real-time continuous monitoring of cell viability, as the assay itself interferes with cell viability [12]. As an alternative to the classic cytotoxicity methods, electrode-impedance-based methods have emerged as a powerful label-free analytical tool to assess cell characteristics [13, 14], including cell viability [15], adhesion, cycle, metastasis, migration, and invasion.

Mass sensors based on micro- and nanomechanical resonators represent a class of ultra-sensitive sensors with enormous potential in the biomedical field [16], with the capability of weighing single cells and single nanoparticles in fluids [17]. Mechanical biosensors have been widely used for ultrasensitive detection of pathogens [18], and also some work has attempted to dynamically inspect living cells [19–24]. There is also some recent work which addresses dynamic (>1 h) qualification of cell viability by a micromechanical mass sensor [25].

Here we report on a micromechanical mass-sensing platform for label-free continuous monitoring (4–5 h) of intoxication in terms of loss in cell adhesion by using the oscillating cantilever of an atomic force microscope (AFM) as probe (more details see in the Additional file 1). AFM is a powerful tool to measure very small forces between a cantilever tip and a surface on the nanoscale, even if the surface to be inspected is soft and submerged in a liquid, e.g., cells in solution. With AFM binding forces between two molecules [26], adhesion of molecules to surfaces [27], adhesion of cells to surfaces [28], or cell to cell adhesion [29] can be recorded. As AFM also allows for lateral resolution also local properties of cell surfaces can be raster-scanned, such as topography [30], localization of adhesion sites [31], local electro-mechanical signaling [21], or local viscoelastic properties [32, 33]. In the following, an assay will be described, in which cell detachment from the cantilever of an AFM is recorded. Hereby the loss of cell adhesion upon cellular exposure to toxic agents, e.g., NPs or chemicals, is monitored.

In general, normal cells could initiate cell death when lost of cell attachment/contact to the extracellular matrix (ECM) occur [3, 34]. Indeed, it is known that disruption of cell adhesion and cell–matrix interactions with successive detachment of cells may be related to signs of cell death [35]. The importance relays on the fact that cell attachment to ECM plays a central role in cell physiology for instance in cell morphology, proliferation, motility among others [36]. Therefore, in the present study we presented a complementary method to monitor loss of cell detachment, a fast-screening-technique to assess dose dependent toxicity using AFM based methodology. The results obtained with this methodology could possibly be associated to early sign of cell death (before cell death is perceptible). To demonstrate the feasibility of the approach proposed, as control, the results obtained from the cantilever were associated with the results obtained with a common conventional cell viability test, the resazurin assay.

## Results and discussion

**Fig. 1:**
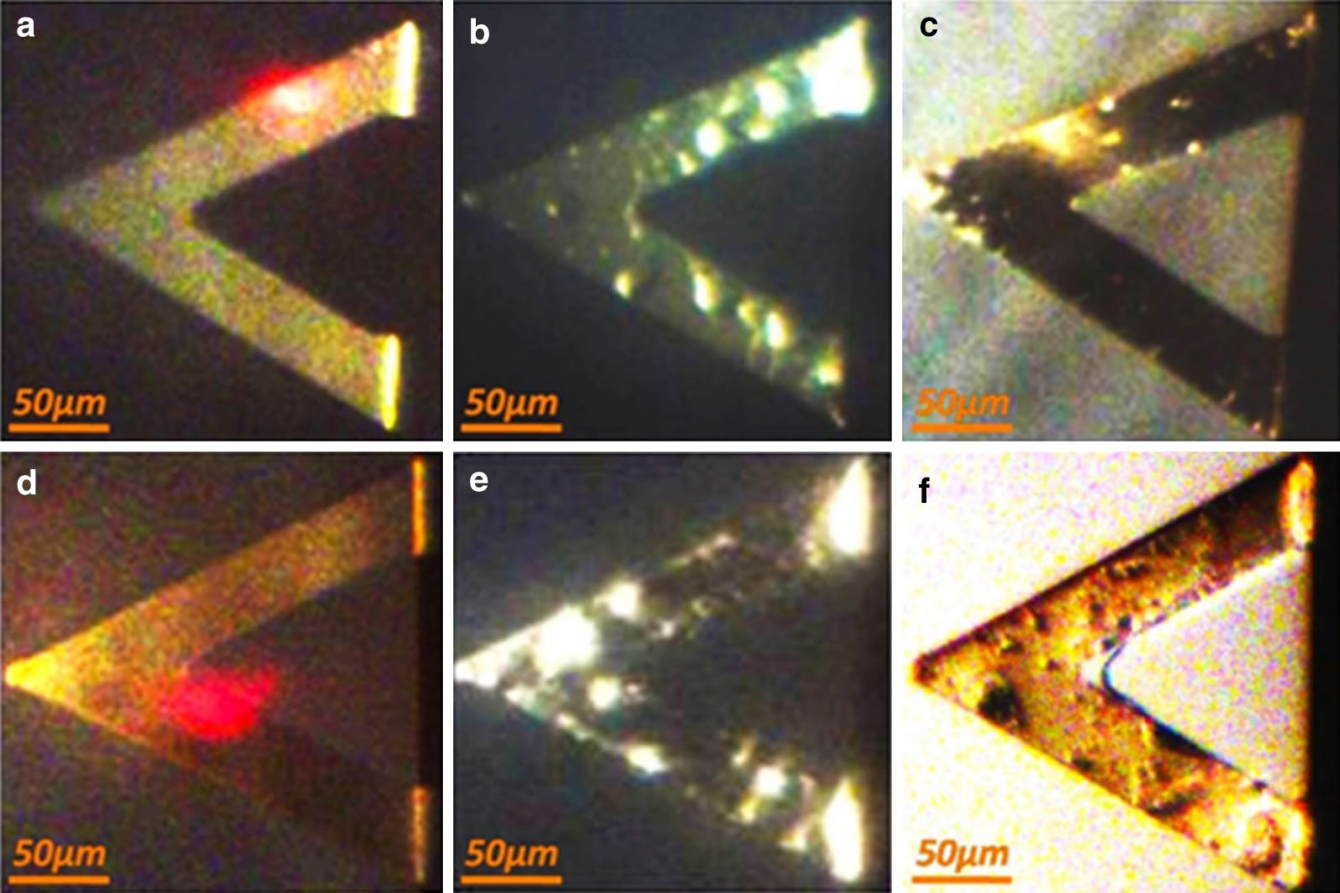
Optical images of one triangular cantilever. **a** The cantilever is oscillating in air before measurement and the surface is found to be clean and flat. **b** The cantilever is oscillating in solution with cells adsorbed. **c** After washing the cantilever with ethanol and rinsing it with PBS most of the absorbed cells desorbed from the cantilever. **d** The cantilever in water is shown before measurement. **e** After injection of Au NPs the cantilever is oscillating in cell medium (DMEM-HG medium, Capricorn Scientific, Ebsdorfergrund) supplemented with 10% fetal bovine serum (Sigma Aldrich). **f** The cantilever is oscillating with absorbed cells and Au NPs in air after measurement without washing

**Fig. 2:**
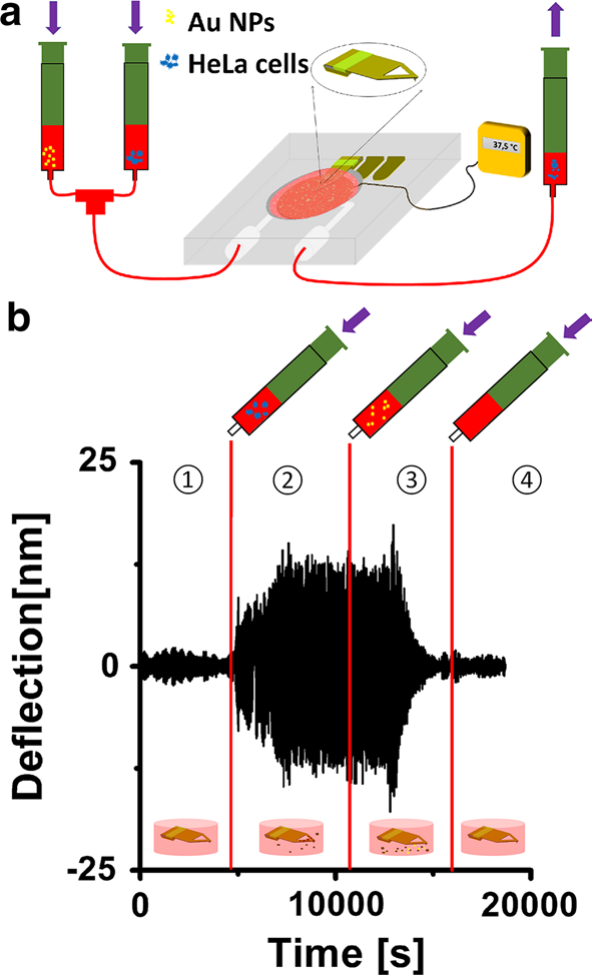
Sketch of the analytical system and the measurement principle. **a** The thermostatic controlled and sealed sample cell houses the AFM cantilever stage. It is equipped with in- and outlets for liquids. Syringes were used as reservoirs for cells and Au NPs. **b** (*1*) The cantilever oscillates at a given frequency and the deflection is recorded over time. The sample cell is fully filled with cell medium and the system is allowed to equilibrate to an approximately constant amplitude. (*2*) Then, cells are injected and allowed to adhere to the cantilever (deflection increases as cells attach, i.e., mass increases). (*3*) Then, after 3600 s, NPs or other chemical agents, whose effect on cell adhesion is to be tested, are injected. After a lag phase, in which NPs or other agents start interacting with cells, the deflection decreases, because more and more cells detach from the cantilever. (*4*) Finally the cantilever is washed with ethanol (70%) and PBS and then, cell medium is injected to regain the initial amplitude and get ready for the next measurement cycle

In our method, a triangular cantilever (SNL-10, k = 0.12 N/m, f_0_ = 23 kHz, Bruker Co) is mounted in a chamber with controlled equilibrated temperature, which can be flushed with different solutions (e.g., NPs or chemical agents in different media and concentrations). Optical images of the cantilever at individual steps of the experiment are shown in Fig. 1. The injection system is schematically shown in Fig. 2a. For a given frequency the cantilever amplitude is highly dependent on the mass of the cantilever or, in our case, on the mass of the cantilever with cells attached (details see in Additional file 1). Because cells attached to different position on the cantilever can have different impact on the deflection, finite elements model can be used for extending the theoretical results of the triangular cantilever [37] (description of finite element model about triangular cantilever in Additional file 1) and in order to control the eventual variations due to cells, the cantilever preparation and characterization are performed for each experiment, i.e., calculation of spring constant before and after experiment, determination of the resonance frequency and deflection sensitivity, to identify that the variance of the deflection is the most appropriate means of analyzing and comparing the data from the different experiments. Figure 2b schematically depicts the method by showing the successive steps through which the cantilever’s dynamic deflection was recorded: (1) The readily mounted cantilever started oscillating in air and then flooded with cell medium, meanwhile the deflection will remain at the initial level. (2) A cell suspension (120 µL of a solution of human cervical cancer HeLa cells at 10^5^ cells/mL) was injected into the sample chamber and was left for ca. 1 h to allow the cells to sediment and eventually attach to the surface of the cantilever [38]. During this time, the deflection amplitude increased due to the added cell mass. (3) In order to study the effect of chemical agents or NPs on cell adhesion, cells were exposed to these agents/NPs at different concentrations. Upon impairment of cells by these substances, cells could lose contact to the AFM cantilever, and the effects on cell adhesion could be monitored and evaluated. Cell detachment is visible as change of mass of the cantilever-cell system. (4) Finally, the cell is flushed with 70% EtOH and PBS buffer to remove all cells and prepare the system for the next measurement. After the rinsing step, the cantilever is optically inspected to confirm that no rest from the previous experiment were present, which is also confirmed by the reset of the real-time deflection to the initial equilibrium (cf., the deflection of pure medium and PBS in Additional file 1).

As a proof of concept, cells were exposed to differently sized and coated gold nanoparticles (Au NPs), as well as other toxic agents, such as ethanol (70%), CdCl_2_, and staurosporine (STS) as a common agent typically used to trigger apoptosis [39]. The effects on cell adhesion upon exposure to NPs and chemical agents at different concentration and time points, were evaluated using the above described setup. Au NPs were used as a NP model in this study because they are interesting materials for biomedical application [40] and thus their biocompatibility needs to be further studied. For instance, it has been reported that the metallic surface of Au NPs could trigger catalytic reactions and cause generation of reactive oxygen species (ROS) [41]. Generation of ROS could damage cellular process and induce ROS associate-toxicity through several mechanisms. Indeed, oxidative stress induced for NPs have been shown. Those cell responses need to be taken in consideration when evaluating possible cytotoxicity effects induced by NPs [42, 43].

In the present study, the effects of three different types of Au NPs with different surface coating and size were evaluated. In general, parameters such as the organic coating around the NPs (e.g., intended as result from synthetic surface modification, or non-intended as result from the absorption of macromolecules from the cell media), size, shape, dose, among others, will affect the impact of the NPs on cell function and morphology, typically by impairing metabolic activity, mitochondrial function, as well shaping the degree and pathway(s) of NP internalization by cells [44].

In the present study we used Au NP suspension having varying NP concentrations (3–400 nM). In Fig. 2b, the AFM data after injection of 50 nM of Au NPs into the sealed and temperature-controlled (37.5 °C) sample chamber is shown. Generally, Au NPs are internalized by cells by different mechanisms, one of the most common pathway is endocytosis [45]. After a lag-phase of ca. 1 h, a time that is typically sufficient for internalization of some Au NPs, a diminishing dynamic amplitude in the AFM signal was observed, resulting in loss of cell adhesion which we ascribed to onset of cytotoxicity. In fact, upon exposure of cells to a potentially dose-dependent toxic agent, cells may change their adhesion properties and be gradually detached from the oscillating cantilever, which would be accompanied by the decrease of the cantilever amplitude, and in this manner recorded. In order to regenerate the cantilever in situ 150 mL of a solution of ethanol (70%) and PBS buffer were injected, respectively. 70% ethanol is known to kill cells. PBS then washed the remaining cell debris away, thus clearing the cantilever, and reduced the amplitude of the cantilever oscillation to the initial value. This process was repeated twice, so that the cantilever will be clearly rinsed and then more measurements may be accomplished during a single session with cells from the same batch. Just before the next measurement and in particular, before cells were injected to the measuring chamber, cell medium was injected again, in order to keep the chamber in conditions suitable for cell culture. The current deflection accompanied with optical images is used for checking the state of the cantilever and chamber.

**Fig. 3:**
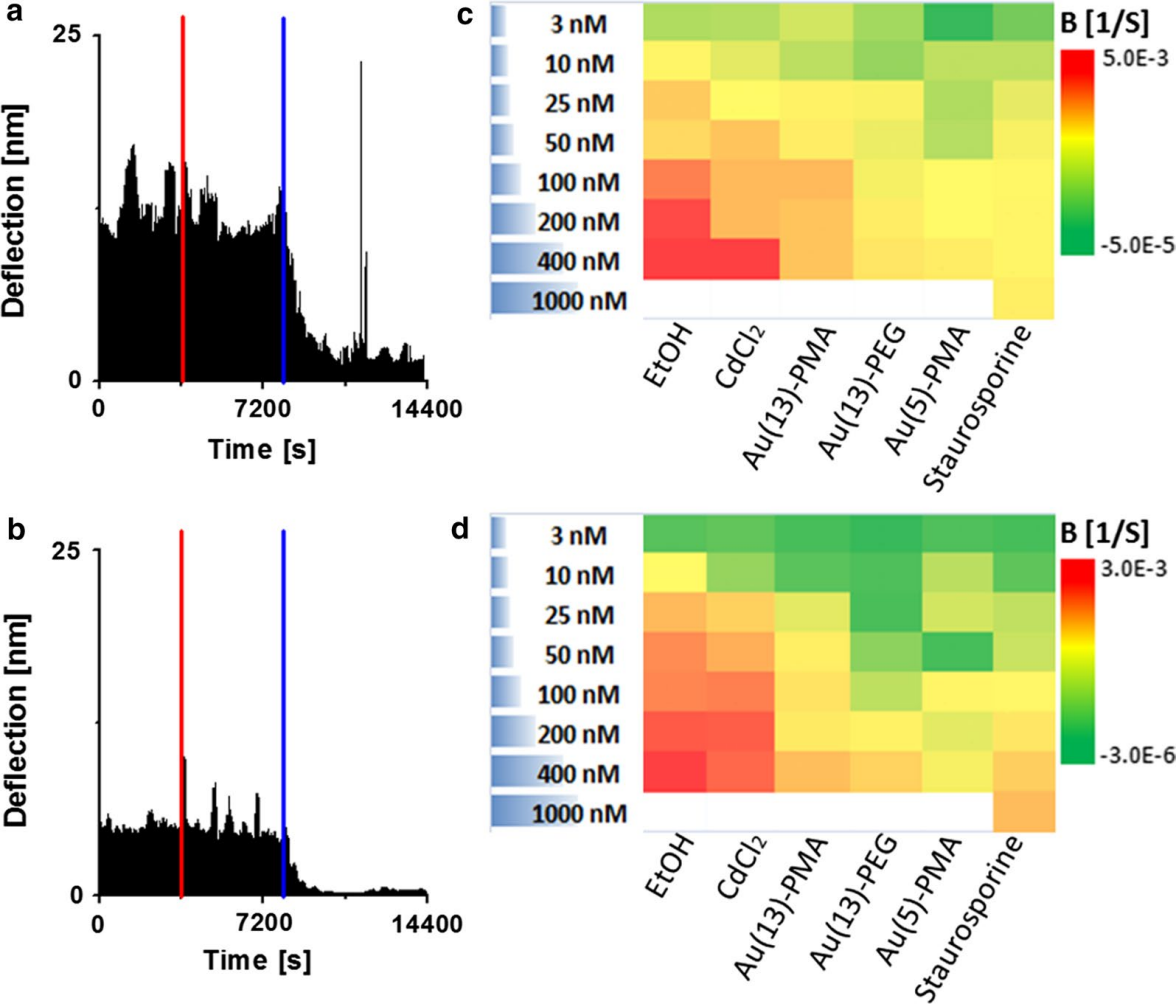
Real-time recorded deflection of cantilever oscillation and analytic results of **a** HeLa cells and **b** MCF7 cells exposed to Au-NP and other toxic chemicals. **a**, **b** Plotted is deflection versus time for HeLa and MCF7 cells exposed to Au(13)-PMA NPs. The time point of injection of the NPs (3600 s) is indicated by the* red line*, and the onset of cell detachment is indicated by the *blue line*, which was automatically set the time point from where the decay of oscillation amplitude was calculated for each measurement. **c**, **d** Heatmaps of the damping constants for HeLa and MCF7 cells as derived from the different measurements for various agents at increasing doses

**Fig. 4:**
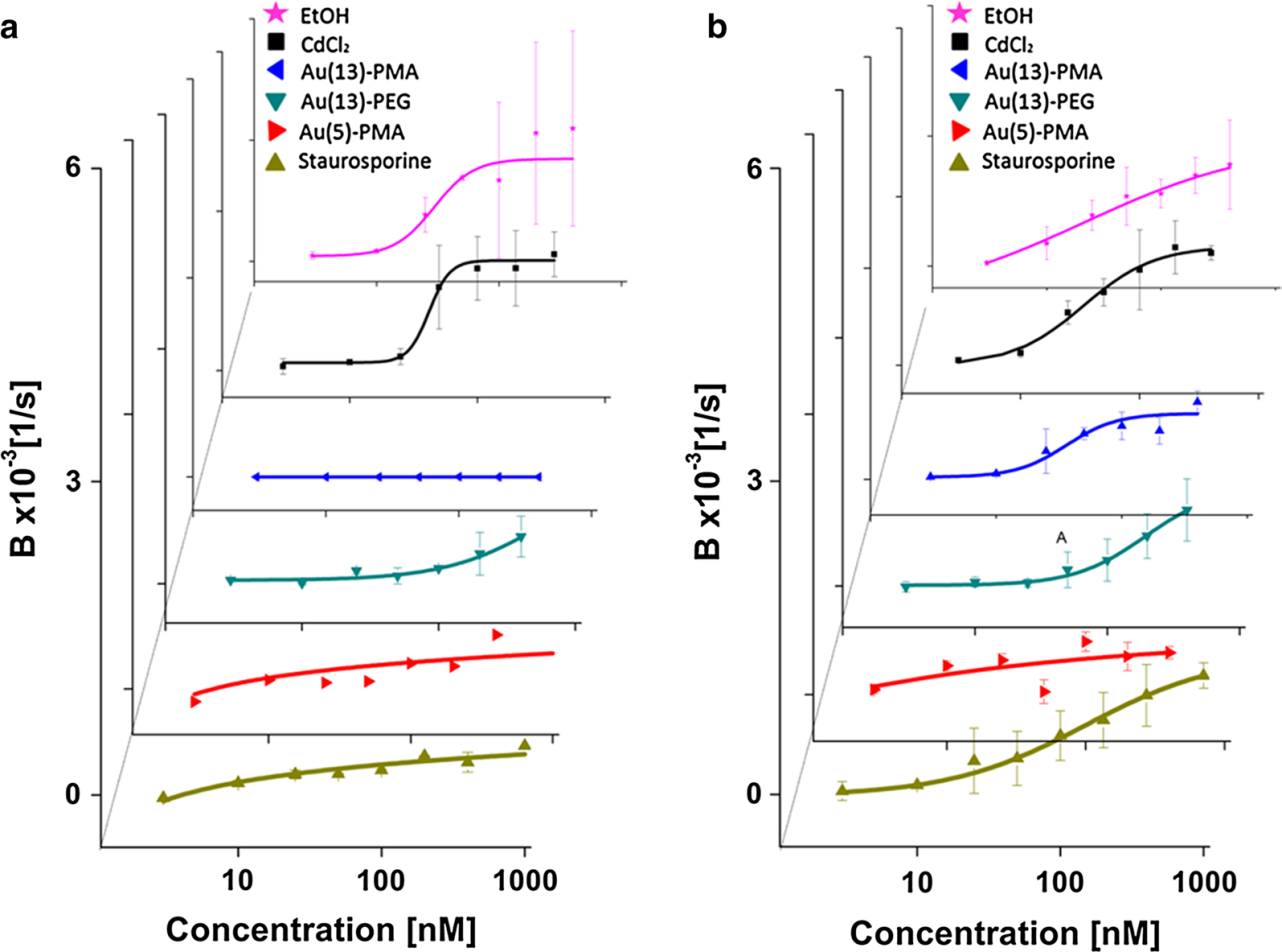
Damping constants B for different agents (a list of all mean values and standard deviations is presented in the Additional file 1) and the corresponding logistic fit curves. **a** Results for HeLa cells are presented, from which based on the respective logistic fit curves the following “half-detachment-dose” values were extracted: 29 nM (EtOH), 43 nM (CdCl_2_), 53 nM (Au(13)-PMA), 640 nM (Au(13)-PEG), 98 nM (Au(5)-PMA), and 78 nM (staurosporine). **b** Results for MCF7 cells, from which based on the respective logistic fit curves the following “half-detachment-dose” values were extracted: 21 nM (EtOH), 33 nM (CdCl_2_), 53 nM (Au(13)-PMA), 190 nM (Au(13)-PEG), 81 nM (Au(5)-PMA), and 150 nM (staurosporine)

For a more detailed and comprehensive evaluation of the presented method, the effects of three different types of Au NPs on cells were investigated (details can be found in “Methods”): (i) Au(5)-PMA, i.e., Au NPs having a core diameter of 5 nm which are grafted with poly(isobutylene-alt-maleic anhydride) dodecylamine (in the following referred to as PMA); (ii) Au(13)-PMA, i.e., Au NPs having a core diameter of 13 nm coated with PMA [46]; (iii) Au(13)-PEG, i.e., Au NPs having a core diameter of 13 nm coated with polyethylene glycol (PEG) [47]. Unless otherwise specified a concentration range from 3 to 400 nM (in terms of NP concentration) was tested. For comparison, other common toxic agents were used, such us ethanol (70%), CdCl_2_, and STS (3 nM–1 µM). Two different cells lines were used for those studies, the human cervical cancer cells (HeLa) and the breast adenocarcinoma cells (MCF7). The dynamic effects on cells caused by the NPs or the chemical agents were monitored by the deflection versus time curves shown in Fig. 3a, b. The agents were injected to the cantilever at different concentrations. Then, after about 1 h exposure, the measurement started (indicated by the red line). After a lag phase, which depended on the agent used, as well as on the dose, cells started to detach (blue line), as indicated by the diminishing deflection amplitude. Notice, that there are no significant changes before NPs have been added, i.e., during the 1 h prior to injection of the agents. After that, the oscillation shows an exponential attenuation, described by a damping coefficient, here referred to as damping value B. The B value is thus an indication for the amplitude damping rate, that is, the damping increases with an increase of B (details about derivation in Additional file 1). The B values extracted from the amplitude decay caused by detachment of cells in each case were quantitatively calculated by a home-made program (details in Additional file 1) (cf., Fig. 3a, b). All original data showing the whole dynamic process for different agents, times and doses are shown in the Additional file 1. Taken these data together, the different B values are condensed into the heatmaps shown in Fig. 3c for HeLa cells and Fig. 3d for MCF7 cells. The following results can be extracted from these heatmaps: (1) In case the same PMA-coating is used, bigger NPs (i.e. diameter of inorganic core of 5 vs. 13 nm), at the same NP concentration, induce a faster onset of cell detachment. (2) In case the NPs had the same diameter of inorganic core (13 nm) and similar surface charge, but different organic coatings (PMA versus PEG) were used, cell detachment is less pronounced for the PEG-coated NPs, probably due to less efficient internalization, as expected from such coatings [48]. (3) Ethanol (necrosis-trigger agent), CdCl_2_, and staurosporine (apoptosis-trigger agent) were used as references in order to underline the general applicability of the method, and to demonstrate that it is not limited to detecting cell detachment due to presence of NPs. As expected, ethanol and CdCl_2_ show early and very fast cell detachment indicative of efficient necrotic agents, while STS shows late and slow cell detachment indicative of apoptosis [49]. The B values versus concentration data points were fitted with logistic curves for both cell lines (cf. Fig. 4), yielding a “half-detachment-dose” value for each agent, so that trends can be extracted. In order to verify that our method could be used to detect and measure toxicity of agents to cells for reference, we evaluated the effects of NPs and compounds exposed to HeLa and MCF7 cells on their cell viability. For that, we used a common cell viability method, the resazurin assay, used to evaluate the metabolic activity of the cells (cf., data in the Additional file 1). This cell viability method was used as a reference control method to compare with the cantilever measurements obtained. As the AFM measurements were carried out without CO_2_ control, the resazurin assays were carried out in the presence or absence of CO_2_ (to mimic the conditions of the cantilever, i.e. without CO_2_, and standard protocols, i.e. with CO_2_). Indeed, the same toxicity trends were observed for the resazurin as for the AFM measurements. There is however one advantage of the AFM assays. In the resazurin assays measurements for different time points have to be carried out separately, while the AFM assays in principle allows for continuous real-time recording.

## Conclusions

The results obtained in this work suggest that the presented method is a generally applicable fast-screening-technique based on label-free real-time monitoring tool, which uses cell detachment from an oscillating cantilever to measure cell intoxication. After desired exposure time, the release rate of cells (as quantified in terms of damping values B) from the cantilever was extracted. We speculate that in future, this method may be applied even to single cells or other cell types such as primary cultures.

## Methods

### Synthesis of Au nanoparticles

#### Synthesis of 13 nm Au nanoparticles

Citrate-capped Au nanoparticles (NPs) with an average inorganic diameter of 13.5 nm (±0.8 nm), as determined by transmission electron microscopy (TEM), were synthesized by largely following the protocol reported by Schulz et al. [50]. Briefly, 144 mL of Milli-Q water was added to 250 mL three-necked round-bottomed flask and heated up until boiling with a heating mantle. First, a mixture of sodium citrate (3.5 mL; 60 mM) and citric acid (1.5 mL; 60 mM) was added to the flask and kept under vigorous stirring for 30 min (450 rpm). A condenser was utilized to prevent the evaporation of the solvent. Then 100 μL of ethylene diamine tetraacetic acid (EDTA 30 mM) was added, followed by 1 mL of 25 mM hydrogen tetrachloroaurate (III) aqueous solution. After ca. 70 s the color of the mixture changed from pale yellow to wine-red, which is indicative of the growth of the Au NPs. In this moment the heating was switched off, but not the stirring. When the temperature of the mixture had dropped down to 95 °C, the flask with the NPs was immersed in ice in order to stop the reaction. The absorbance at 450 nm [extinction coefficient ε(450) = 1.6 × 10^8^ M^−1^ cm^−1^] was used to determine the concentration of the NPs, as previously described by Haiss et al. [51].

#### Synthesis of 5 nm Au NPs

A modified protocol of the two-phase method published by Brust et al. and Holz et al. was used to produce tetraoctylammonium bromide-capped Au NPs with an inorganic diameter of 5.5 nm (±1.0 nm), as determined by TEM [52, 53]. Briefly, at room temperature, an aqueous solution of hydrogen tetrachloroaurate-(III) (40 mM, 25 mL) and a solution of tetraoctylammonium bromide (TOAB) in toluene (50 mM, 80 mL) were mixed and vigorously shaken (ca. 5 min) in a 500 mL separation funnel. Then, once the AuCl_4_ ions were fully transferred into the toluene phase, the organic phase was transferred into a 250 mL round bottom flask. Then, a freshly prepared aqueous solution of NaBH_4_ (350 mM, 25 mL) was added to the solution of gold precursors in toluene under vigorous stirring and kept under stirring for 1 h. The solution was then transferred to a 500 mL separation funnel and 25 mL of 10 mM HCl was added to remove the excess of NaBH_4_. The mixture was vigorously shaken and the aqueous phase was discarded. Then 25 mL of 10 mM NaOH were added to remove any excess of acid, followed by four washes with Milli-Q water (25 mL). The toluene phase containing the Au NPs was transferred to a 250 mL round bottomed flask. Then, the solution was left under stirring overnight at room temperature. Then, original TOAB coating was exchanged by 1-dodecanethiol, by mixing (65 °C, 3 h) the original NP dispersion in toluene with a solution of 1-dodecanethiol in toluene (4.17 M, 10 mL). Then, the 1-dodecanethiol-capped Au NPs were purified from agglomerates by centrifugation at 1 × 10^3^ *g*, whereby the NPs remained in the supernatant. To remove the excess of 1-dodecanethiol, the NPs were precipitated by addition of methanol and collected by centrifugation (1 × 10^3^ *g*). The washing step with methanol was repeated three times to minimize the presence of free surfactant. In order to calculate the concentration of NPs, the absorbance at 520 nm [extinction coefficient ε(520) = 8.7 × 10^6^ M^−1^ cm^−1^] was used, as previously reported [54].

### Surface modification of Au NPs

#### PEGylation of 13 nm citrate-capped Au NPs

To 150 mL of the as prepared citrate-capped NPs (NP concentration ca. 1.8 nM), 2.7 mg of α-thio-ω-carboxy poly(ethylene glycol) (HS-PEG-COOH, M_W_ = 987.19 Da from Iris Biotech) were added, equivalent 10^4^ PEG molecules added per NP. Thus, sufficient PEG was added to ensure full PEG saturation of the NP surface. The PEGylated Au NPs were purified from PEG excess and re-suspended in deionized water by centrifugal precipitation (three times at 15 × 10^3^ *g*, 30 min).

#### Polymer coating poly(isobutylene-alt-maleic anhydride) dodecylamine-grafted, in the following referred to as PMA of 13 nm citrate-capped Au NPs

Citrate-capped Au NPs were transferred from aqueous media to organic solvent following the protocol of Soliman et al. [46]. Briefly, 3·10^4^ PEG molecules (M_W_ = 750 Da; α-methoxy-ω-mercapto-poly(ethylene gylcol) (HS-PEG-CH_3_O) from Rapp Polymere) per NP were added and kept under vigorous stirring for 2 h. Then, a 0.4 M solution of dodecylamine (DDA) in chloroform (equal volume as the aqueous solution of NPs) was mixed with the NPs under vigorous stirring, which ultimately allows to transfer the NPs from the aqueous to the chloroform phase. A small amount of NaCl (50 μL 2 M) was added to speed up the NPs’ phase transfer. The NPs were then cleaned twice by centrifugal precipitation (8960*g*) from excess of PEG and DDA. The precipitated NPs were collected and dispersed in chloroform, in which their concentration was determined by UV/Vis spectroscopy with the molar extinctions coefficients as provided above. Yet, to get PMA-coated Au NPs colloidally stable in aqueous solution, the Au NPs previously coated with PEG/DDA were coated with the amphiphilic polymer PMA by largely following the protocol described by Lin et al. [55]. Briefly, 75% of the anhydride rings of poly (isobutylene-alt-maleic anhydride) were modified with DDA by mixing in tetrahydrofuran (THF) at 65 °C under stirring (12 h). The modified polymer (i.e., PMA) was dried using a Rotavapor at 40 °C under reduced pressure and dispersed in 30 mL chloroform, yielding a stock solution with a final PMA concentration of 0.75 M. Notice that 0.75 M refers to the concentration of the monomers of poly(isobutylene-alt-maleic anhydride). Then, to efficiently achieve PMA-coating of the NPs, a specific volume of PMA, which depends on the total effective surface area (A_eff_) of the NPs, was used as described by Soliman et al. [46]. Briefly, the NPs were mixed with PMA (0.75 M in terms of monomer units; R_p/Area_ = 3000, where R_p/Area_ refers to the number of PMA monomers added per nm^2^ of A_eff_) in a round flask and diluted with chloroform. After 25 min, the chloroform was slowly evaporated at 42 °C under reduced pressure using a Rotavapor, until the solvent was completely evaporated. This procedure was repeated twice. Finally, the dried product was dissolved in sodium borate buffer (SBB, pH = 12), which hydrolyzed the maleic anhydride groups of the PMA, yielding carboxyl groups and thereby providing the NPs with colloidal stability in aqueous solution. The PMA-coated NPs were then filtrated through a syringe membrane filter (0.22 μm pore size). Finally empty micelles formed by PMA and excess of free PMA were removed by precipitation of the PMA-coated NPs using centrifugation (8960*g*; 40 min, twice) and the buffer was exchanged to water.

#### PMA coating of 5 nm Au NPs

Equivalently, PMA coating was carried out as described for the 13 nm NPs. A value R_p/Area_ = 150 PMA monomers per nm^2^ was used instead, which was experimentally optimized to warrant for colloidal stability of NPs with about the same size of inorganic core. The PMA-coated NPs were purified first by gel electrophoresis, as described in previous works (e.g., see Lin et al. [55]) and then by ultracentrifugation (150 × 10^3^ *g*; 60 min, three times).

### Characterization of NPs

Transmission electron microscopy (TEM), UV–Vis spectroscopy, dynamic light scattering (DLS) and laser Doppler anemometry (LDA) were used to analyze the colloidal properties of the NPs.

#### TEM imaging

TEM images of the samples were acquired in a JEM-1230 transmission electron microscope equipped with a LaB6 cathode running at 120 kV and an ORIOUS SC1000 4008 × 2672 pixels CCD camera (Gatan UK, Abingdon Oxon, UK). UV–Vis spectra were obtained with an Agilent 8453 spectrometer. DLS and LDA measurements were carried out with a Malvern Zetasizer. In Additional file 1, Figure S1a, c show TEM micrographs of PEGylated 13 nm Au NPs and PMA-coated 5 nm Au NPs with negative staining, in which a PEG layer (thickness of ca. 5 nm around cores of 13 nm) and the PMA-coating (thickness of ca. 5 nm around the 5.5 nm cores) are clearly discernible. Additional file 1: Figure S3b shows a TEM micrograph of the PMA-coated NPs (here, only the diameter of the Au core gives contrast).

#### TEM negative staining

Uranyl acetate was used as negative stain, which allows the formation of a uniform, consistent, and high contrast staining. The sample was prepared on carbon film 400 copper mesh grids purchased from Electron Microscopy Sciences (Hatfield, USA). The specimen grids were exposed to glow-discharge treatment under air plasma for 20 s (2.0 × 10^−1^ atm. and 35 mA) using a MED 020 modular high vacuum coating system (BAL-TEC AG, Balzers, Liechtenstein). Negatively charged carbon grids were used within 5 min after treatment to ensure hydrophilicity. The on-grid negative staining was performed using a slightly modified single-droplet negative-staining procedure. 1.5 μL sample droplet of NP concentration ranging from 6 to 15 nM followed by three 2.5 μL droplets of 0.25% weight/volume (w/v) uranyl acetate aqueous solution were placed on a clean Parafilm piece. The treated grid was incubated on the sample droplet for 1 min and then on the staining droplets for 3, 3, and 60 s, respectively. After each incubation step the excess fluid was nearly fully removed by touching the grid edge with Whatman filter paper. Finally, the sample was fully dried for 20 min at 2.0 × 10^−1^ atm.

#### UV–Vis absorption spectroscopy

The UV–Vis absorption spectra of the three polymer-coated samples are shown in Addtional file 1: Figure S3d, which clearly show the surface plasmon resonance band of the colloids (ca. 520 nm), more intense in the case of the 13 nm NPs, as expected.

#### Zetasizer measurements

DLS and ζ-potential values of the three samples are summarized in Table 1. The hydrodynamic diameter (d_h_) of the PEGylated Au NPs, as determined by DLS, yielded 22 nm, which matches very well the observations by negative staining TEM. Note however, that the DLS and the negative staining were obtained in aqueous solution and vacuum, respectively. The d_h_ values of PMA-coated 13 and 5 nm Au colloids were 17 and 11 nm, respectively. Sizes as determined by TEM (inorganic core; d_TEM_) and DLS (d_h_), and ζ-potential values of the polymer coated Au NPs are summarized in Table 1.

**Table 1:** Comparison of diameters taken from TEM and hydrodynamic diameters taken from DLS, as well as ζ-potential values of the examined NP samples

Sample	d_TEM_/nm	d_h_(number)/nm	ζ-potential/mV
Au(13)-PEG	13.5 ± 0.8	21.9 ± 0.3	−23.0 ± 1.9
Au(13)-PMA	13.5 ± 0.8	16.7 ± 0.4	−20.2 ± 0.8
Au(5)-PMA	5.5 ± 1.0	15.3 ± 0.8	−42.7 ± 1.3

DLS and ζ-potential data were recorded in MilliQ water. The hydrodynamic diameter corresponds to the mean value ± standard deviation as obtained from the number distributions

### Reference assay

#### Cell culture

HeLa and MCF 7 cells were obtained from the American Type Culture Collection (ATCC, Manassas, VA). Briefly, HeLa cells were cultured in Dulbecco’s Modified Eagle’s Medium (DMEM) (# D5796) containing 10% Fetal Bovine Serum (FBS) (#S0615), 1% of Penicillin/Streptomycin (P/S) (# 15140-1229) and GlutaMAX™ (#35050-038). MCF7 cells were cultured in Eagle’s Minimum Essential Medium (EMEM) (# M5650 supplemented with 10% FBS, 1% of P/S and 0.01 mg/mL human recombinant insulin (# I3536). The cell cultures were kept at 37 °C in a humidified atmosphere of 5% CO_2_ in air. At confluence, cells were washed with PBS and detached with 0.05% Trypsin EDTA (# 25300-054) solution. Cells then were reseeded in flasks for cell culture or seeded in 96-well plates for the experiments.

#### Cell viability assay

Cell viability of HeLa and MCF7 cells exposed to Au NPs and chemical agents was evaluated by the Resazurin assay [AlamarBlue^®^ (# 765506) Thermo Fisher, Germany] as previously reported [56–58]. For that, HeLa and MCF7 cells were seeded in 96 black polystyrene plates at the density of 10.000 cells/well in complete cell culture media and were incubated overnight at 37 °C, 5% CO_2_. The next day, cells were exposed to NPs and chemical agents at desired concentration for 4 h in the presence or absence of 5% CO_2_ at a final volume of 100 μL per well. After the desired time, cells were washed once with PBS, then 100 μL of 10% resazurin solution (in complete cell media) was added to the cells and incubated for 4 h at 37 °C and 5% CO_2_. The fluorescence intensity was measured for the presence of resazurin and resorufin with a 96-microwell plate reader connected to a fluorometer (Fluorolog-3, from Horiba Jobin–Yvon, Germany) at an excitation wavelength of 560 nm. The emission was recorded in the range of 570–650 nm, of which an integrated fluorescence intensity was determined. This integrated fluorescence intensity was considered to be proportional to cell viability. Cell viability was normalized to 100% for untreated cells. The results are presented as mean cell viability ± the respective standard deviation (SD), as obtained from three independent experiments (e.g. cell cultures), each one performed in triplicate. Upon incubation with high concentration of toxic agents, cell viability is decreased. All diagrams are presented in Additional file 1.

## Additional file

**Additional file 1.** Additional information.
