# Supplementary material for: Real-time, label-free monitoring of cell viability based on cell adhesion measurements with an atomic force microscope
Source: J Nanobiotechnology. 2017 Mar 22;15:23. doi: 10.1186/s12951-017-0256-7 (PMC5361698; doi:10.1186/s12951-017-0256-7)
Supplement: Supplementary file 1 — Additional file 1. Additional information. [file 12951_2017_256_MOESM1_ESM.docx]

**Real-time, label-free monitoring of cell viability based on cell adhesion measurements with an atomic force microscope**

Fang Yang^1^, René Riedel^1^, Pablo del Pino^2^, Beatriz Pelaz^2^, Alaa Hassan Said^2^, Mahmoud Soliman^2^, Shashank R. Pinnapireddy^3^, Neus Feliu^2^, Wolfgang J. Parak^2,4^, Udo Bakowsky^3^, Norbert Hampp^1,5*^

^1^ University of Marburg, Department of Chemistry, Marburg, Germany

^2^ University of Marburg, Department of Physics, Marburg, Germany

^3^University of Marburg, Department of Pharmacy, Marburg, Germany

^4^ CIC biomaGUNE, San Sebastian, Spain

^5^ University of Marburg, Material Science Center, Marburg, Germany

*corresponding author: hampp@uni-marburg.de

**Supporting information**

1. Characterization of Au NPs 2
2. Cantilever deflection upon force loading 3
3. Oscillation system setup 5
4. Scheme of the cantilever 7
5. Baseline correction for analysis of data 7
6. Calculation of damping curve and damping constant 8
7. Raw data of cell-detachment measurements 10
8. Raw data of pure medium and PBS 14
9. Reference Assay data 15
10. Safety precautions when using Staurosporine 21
11. References 21

*I) Characterization of Au NPs*

TEM and UV-Vis characterization of the Au NPs used are shown in Figure S1.

Figure S1: a,c) Negative staining TEM micrographs of samples AuNP(13)-PEG and AuNP(5)-PMA. b) TEM micrograph of sample AuNP(13)-PMA. d) UV-Vis spectra of AuNP(13)-PEG (black line), AuNP(13)-PMA (red line) and AuNP(5)-PEG (blue line).

II) Cantilever deflection upon force loading

The deflection of a cantilever during an atomic force microscope (AFM) measurement is proport­ional to the force that acts on the cantilever and results from interaction of the cantilever with the sample according to Hook’s law:

|  | $\Delta z=k_{f}^{-1}F$ | (1) |
| --- | --- | --- |

where Δz is the deflection, k_f_ the spring constant, and F the acting force, i.e. the loading force. In this work, the cantilever was driven to oscillate harmonically by a piezo element. The deflection of pure harmonic oscillations can be described by Equation 2

|  | $\Delta z=A\cos(2\pi\nu\cdot t+\varphi)$ | (2) |
| --- | --- | --- |

where A is the maximum deflection, ν is the frequency, and ϕ is the phase.

In case a force is applied on a harmonically oscillating cantilever, the total energy of the oscillation, which is proportional to the square of the amplitude A^2^, is affected. Hence, the amplitude variation is highly depending on the loading force, while the oscillation frequency in first order remains constant. Equation 3 shows the dependency of the deflection z on an applied loading force F, caused by the mass of the cells attached, at loading position x within a cantilever of length L^1,2^ (0 ≤ x ≤ L).

|  | $z=\frac{F\cdot x^{2}\left( 3L-x \right)}{6EI}=\frac{g{\cdot x}^{2}\left( 3L-x \right)}{6EI}\cdot m_{cell}$ | (3) |
| --- | --- | --- |

In eq. 3 the deflection z of the bar depends on the elastic modulus E and I (the area moment of inertia), which are characteristic properties of the bar. F is the loading force, in this case due to the mass of cells, to the bar and the position x indicates where the force applies to the bar. This theory is appropriate to treat cells attached on a cantilever, whereby the deflection is varied depending on the different loading forces. Attached cells will cause an increased deflection. Due to loss of cell adhesion the number of cells remaining on the cantilever decreases, which is observed as decreasing deflection amplitude.

A finite element simulation of triangular cantilever is used for explaining that different loading position will have different deflections. The Figure S2 shows displacement of cantilever (color map) induced by the force vector. When the force is applied on the apex of the cantilever, the impact will be maximal.


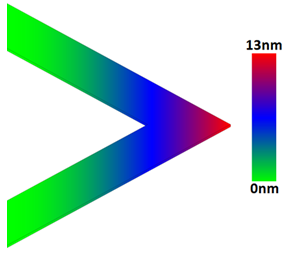


Figure S2: finite element simulation of triangular cantilever. Different color shows different displacement of the cantilever applied by the force vector.

*III) Oscillation system setup*

Figure S3 shows the fluid chamber, which is the core part of the system. It comprises an inlet and an outlet for fluids and a cantilever holder. The cantilever was mounted and medium was injected through the inlet using a plastic syringe. By this way a droplet formed around the cantilever. Deflection of the cantilever was recorded by the AFM. The proportionality factor so called “deflection sensitivity”, which ensures a good linear relation between the voltage applied and the deflection of the spot over the whole area of the screen, should be determined for each measurement, so that loading force of the cantilever can be quantitatively analyzed. Another syringe (Figure S3) was used to remove the fluid through the outlet (blue arrows).


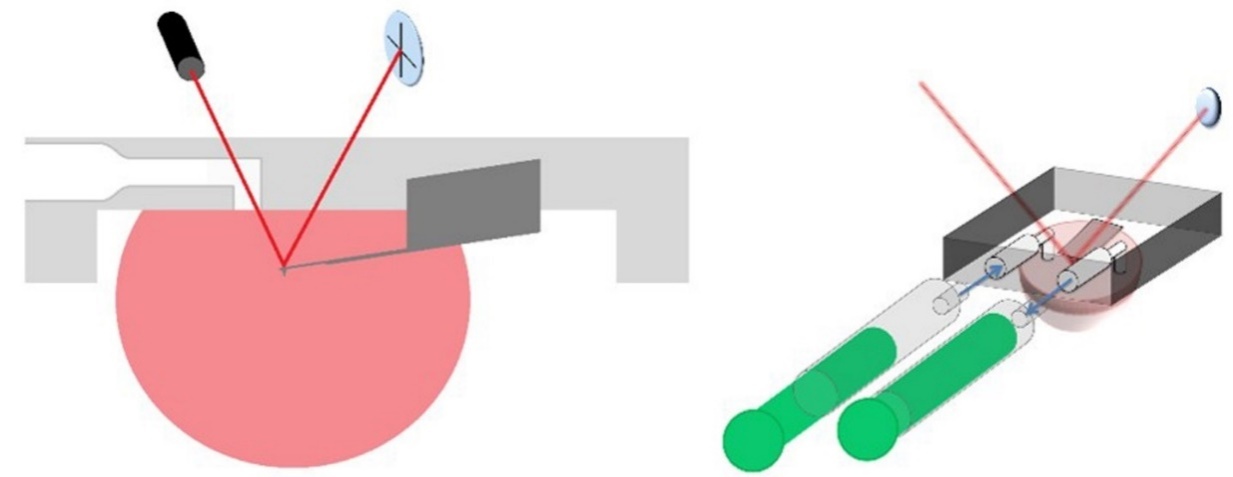


Figure 3: (left) reservoir based on fluid chamber with cantilever, laser beam and photodiode. (right) injecting system using plastic syringes connected to the reservoir.

The fluid chamber inserted into the AFM head was built as a reservoir thermostated to 37 °C. Figure S4 shows the experimental setup. An incubation chamber was used for storing the cells at 37 °C and 5% CO_2_. Atmospheric conditions in the chamber were controlled by a heating plate with circulation fan and CO_2_ controller. The tip deflection was recorded by the AFM picoforce spectrometer connected to a Keithley 2000 multimeter. During the whole measurement the cantilever was imaged by a camera installed on the microscope, so that the actual status of the cantilever could be correlated with the deflection dependent on the number of attached cells.

| 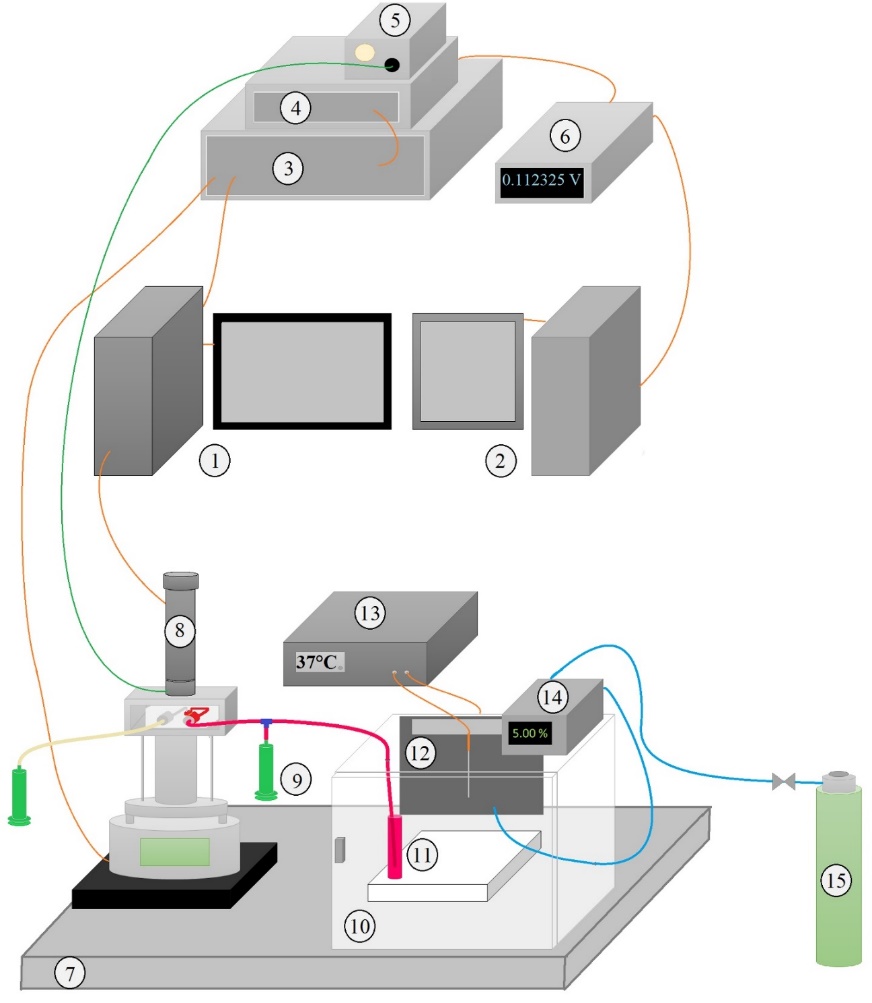 |
| --- |
| Figure S4: Scheme of the experimental setup. (1) Computer with software for recording visual images from the AFM tip. (2) Monitoring system for measurement and data recording. (3) Multimode Controller of the AFM. (4) Picoforce-spectrometer for deflection recording. (5) Fiber-coupled light source for the optical camera. (6) Keithley 2000 multimeter for recording the data from the Picoforce spectrometer. (7) Laser table. (8) Light microscope with a camera for visually observation of the cantilever. (9) Input syringe. (10) Incubation box for cells. (11) Cell storage tubes. (12) Heating plate with circulation fan for keeping a homogeneous temperature. (13) Temperature controller for regulation of the heating inside the incubation box. (14) CO_2_ controller. (15) CO_2_ container. |

*IV) Scheme of the cantilever*

| 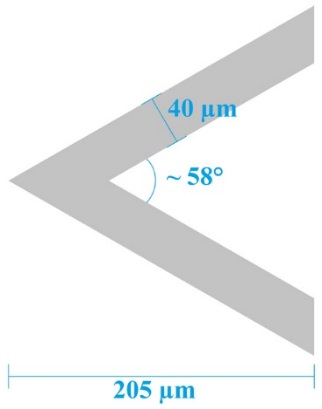 |
| --- |
| Figure S5: Scheme of cantilever SNL-10. |

Figure S5 shows a scheme of a cantilever (SNL-10, k = 0.12 N/m, f_0_ = 23 kHz, Bruker) depicting the area for the cells to attach to.

*V) Baseline correction for the analysis of data*

The baseline of the deflection of the cantilever showed a drift over time due to temperature fluctuations in the chamber, fluid flow in the fluid drop, etc. Thus, the baseline was flattened by subtracting a line obtained by the least square fit method (5^th^ degree function). From all data the calculated baseline was subtracted. Figure S6 shows an example of a measurement before (blue) and after (red) baseline correction.


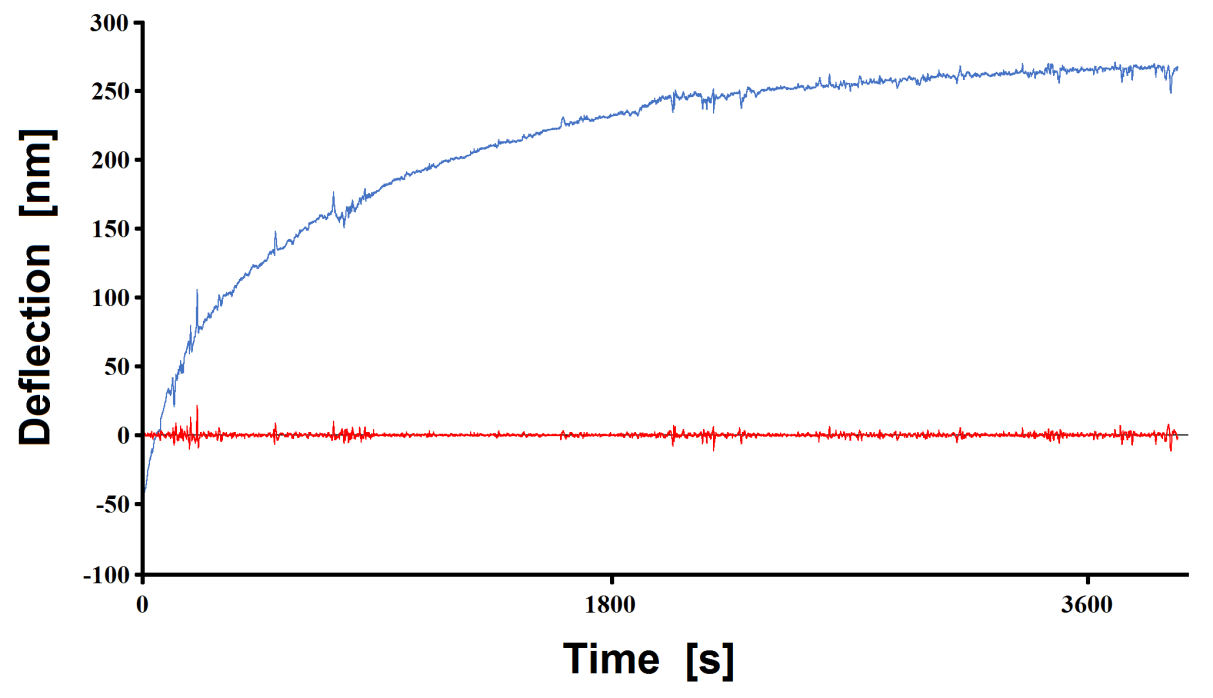


Figure S6: Example of the applied baseline correction of the cantilever deflection trace versus time. The blue data points show the raw measurement data, whereas the red curve represent the calculated data after baseline correction.

VI) Calculation of damping curve and calculation of damping constant

The amplitude of oscillation increases for increasing numbers of cells attached to the cantilever. When a cell is desorbed from the cantilever the amplitude decreases. The damping constant of the oscillation is linked to the detachment rate of the cells. The detachment rate of cells here is used as measure for cell viability. These measurements are done in real-time. In order to minimize noise not related to the cantilever deflection, a sliding average was calculated for each absolute value for a time interval of -1800 s to +1800 s (see red line in Figure S7). The values higher than the curve of the sliding average were not considered for further calculations.


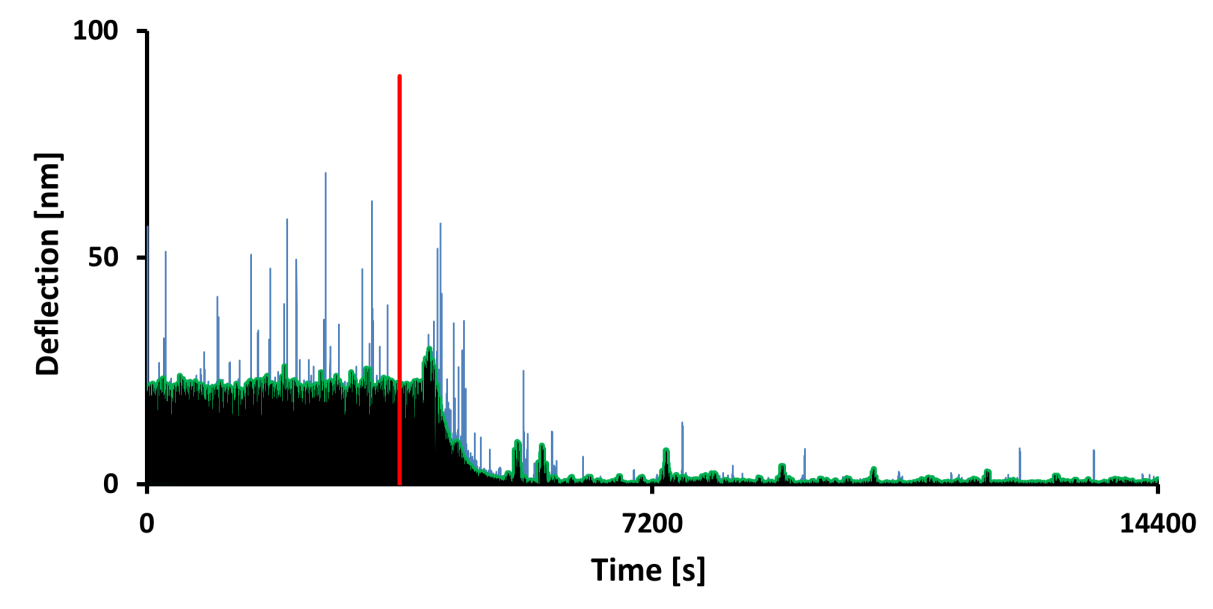


Figure S7: Absolute values of cantilever deflection versus time. HeLa cells were exposed to Au NPs after 3600 sec (vertical red line) of incubation. Outliers (showing in grey) were truncated at the green sliding average line.

The measurements comprise several parts. The first part is from t_1,S_ = 0 s to t_1,E_ = 3600 s. During this period HeLa or MCF7 cells in medium were measured without added Au NPs. After injection of the Au NPs (red line), a certain lag-phase is observed. One may speculate why after a while a small increase in the amplitude is observed. This is in the same range as some random peaks at higher times (e.g., at around 7200 s in Figure S8, Region 1), and thus will not be further interpreted here. However, after exposure to Au NP (at high enough concentrations), an exponential decrease on deflection can be observed. Cells begin to detach and the deflection decreases (Region 2 in Figure S8), until a minimum is reached.


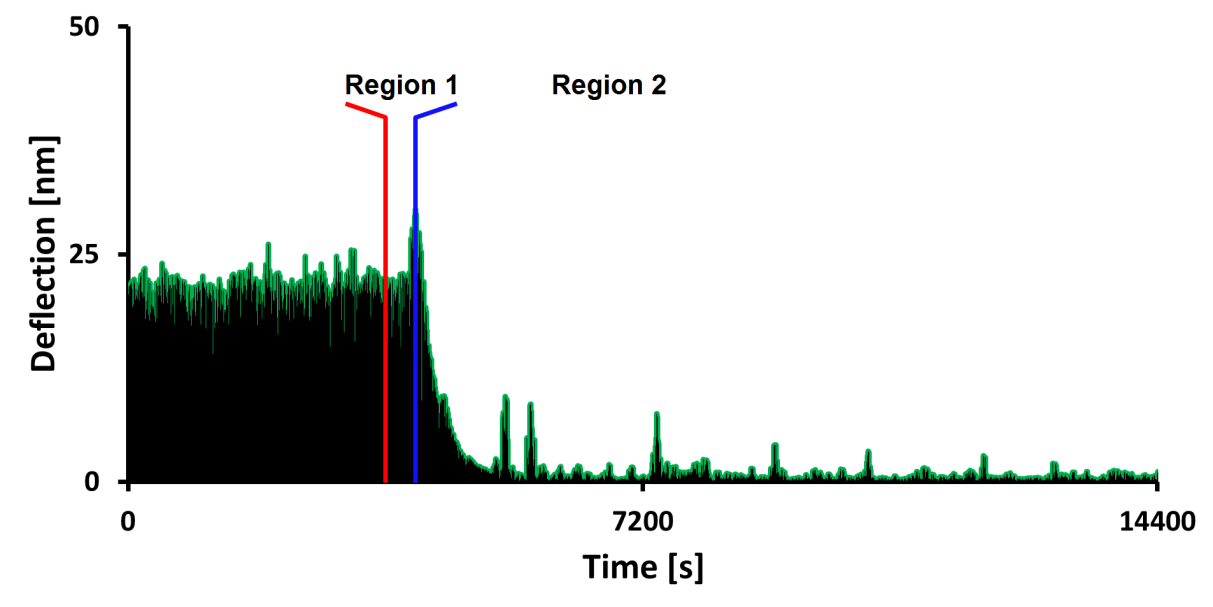


Figure S8: The data shown in Figure S7 (without the outliers) are divided into two parts for further processing. Region 1 (from red to blue line) starts with the addition of the toxic agent, followed by an increment of the deflection. As this is not seen in all measurement using Au NPs (see Figure S9), this is not discussed further here. Region 2 shows the detachment of cells and the accompanied decrease of the amplitude until a constant value is reached.

The damping of the oscillation was approximated by an exponential function (region 2), where A_0_ is the amplitude at t = 0, and e^-Bt^ describes the time-dependent damping of the oscillation.

|  | $A\left( t \right)=A_{0}e^{-Bt}$ | (4) |
| --- | --- | --- |

Resolving this equation for B leads to equation (5) where B is the damping coefficient, which

|  | $B=-\frac{t}{\tau}ln\left( \frac{A}{A_{0}} \right)$ | (5) |
| --- | --- | --- |

characterizes the cell detachment and amplitude decrease rate, respectively. The B value describes the negative logarithmic ratio of the amplitude at point t and the original amplitude (at t = 0), divided by the time. Keeping in mind that A/A_0_ ≤ 1, the B value is thus an indication for the amplitude damping rate, whereas the damping increases with an increase of B. Thus, a high positive B value stands for a high toxic effect to the cells. The B values were calculated using a least square fit of the data points to the function given in eq. (5).

VII) Raw data of cell-detachment measurements

The effect of addition of Au NPs etc. to attached cells was monitored and recorded by curves of deflection versus time. All potentially toxic agents, i.e., ethanol (EtOH) (70%), CdCl_2_, Au(5)-PMA, Au(13)-PMA, Au(13)-PEG, and staurosporine (STS) were measured with HeLa cells and MCF7 cells respectively. The original data are presented below.


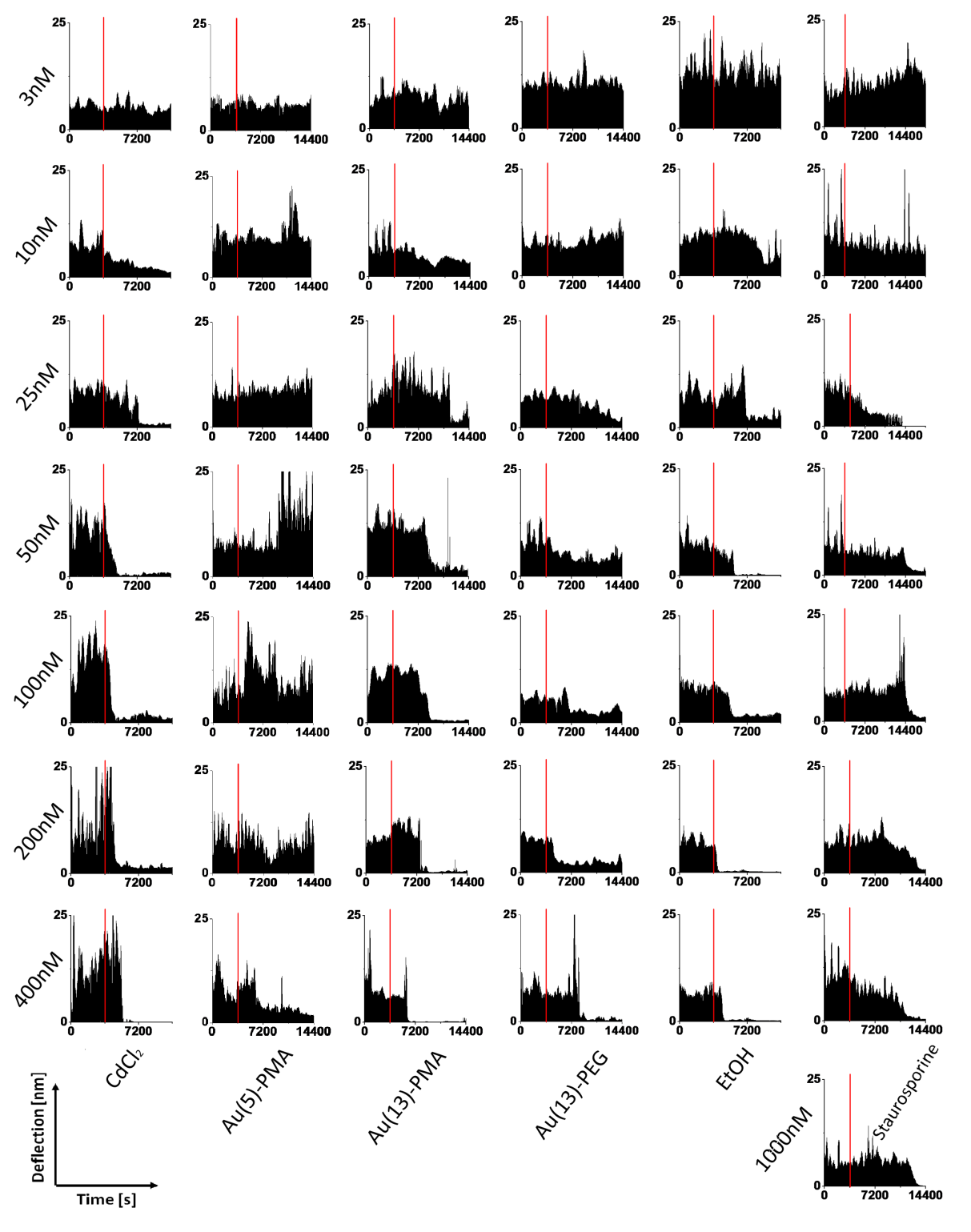


Figure S9-1: Raw data of deflection curves about different agents with different concentration from 3 nM to 400 nM or 1000 nM as recorded with HeLa cells. The red lines indicate the time when the agents were injected to the cells.


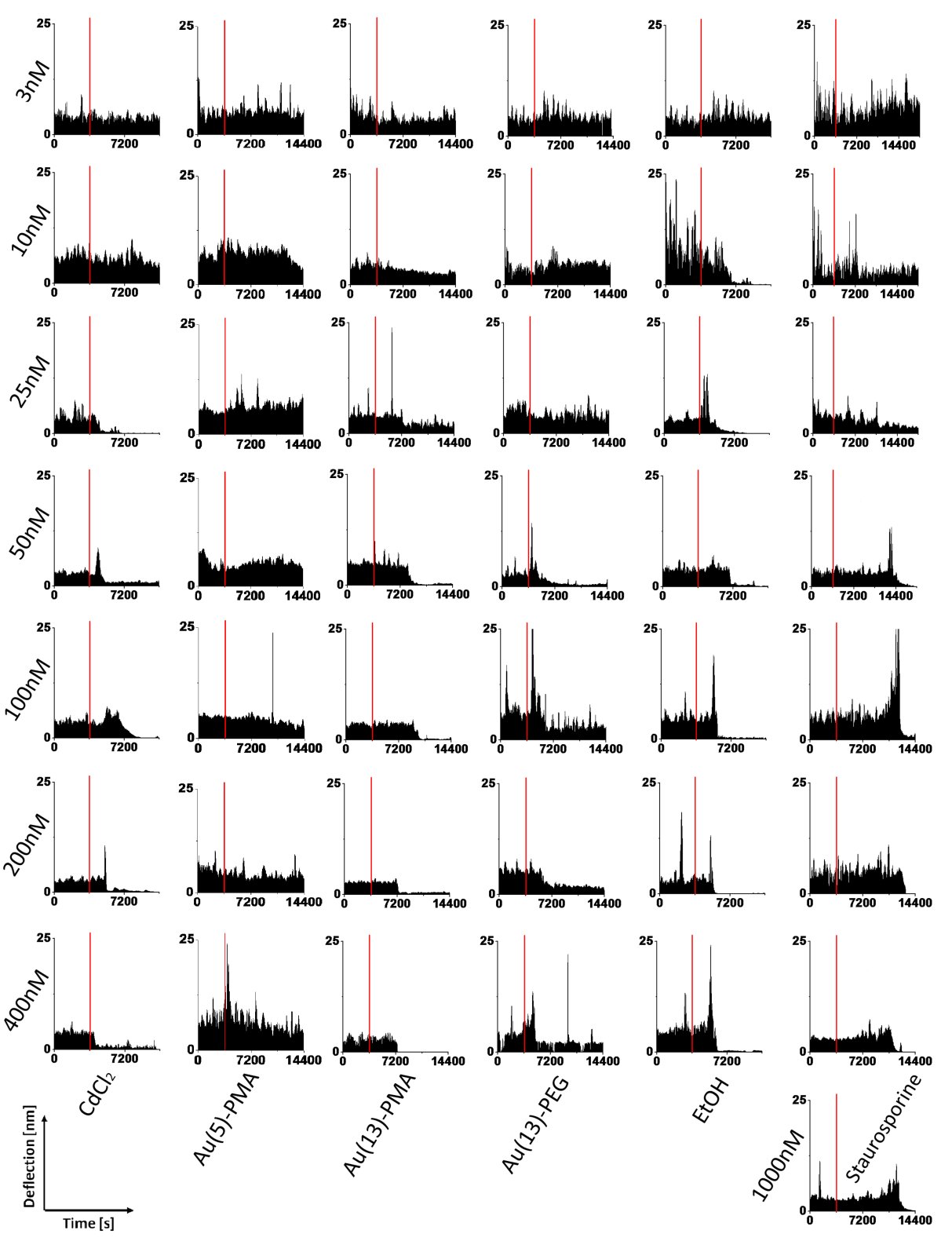


*Figure S9-2: Raw data of all deflection curves about different agents with different concentration from 3 nM to 400 nM or 1000 nM to MCF7 cells. The red lines indicate the time when the agents were injected to the cells.*

According to these diagrams (Figure S9-1 and -2), an increased amplitude can be observed after administration of Au NPs, EtOH and Staurosporine at varying time points after administration (i.e., in a sample- and dose-manner). It typically precedes decrease of amplitude (in a dose-, sample-manner). As it is also observed after adding EtOH or Staurosporine, it cannot be interpreted as an increment of mass due to uptake of Au NPs. We think that it might be due to a trampoline-like effect, by which partially detached cells temporally induce an amplitude increase, just before they are 100% detached. In addition, in the case of Staurosporine it occurs quite later after administration (>2h), before the onset of detachment, and therefore, it cannot be due to an artifact introduced by adding any NPs or chemical agents.

Using a self-written software, the B-values of each trace were calculated. For each series of agents, at least double measurements were done, the mean B‑values for both cell lines upon incubation with different agents were collected and are shown in Table S1 below.


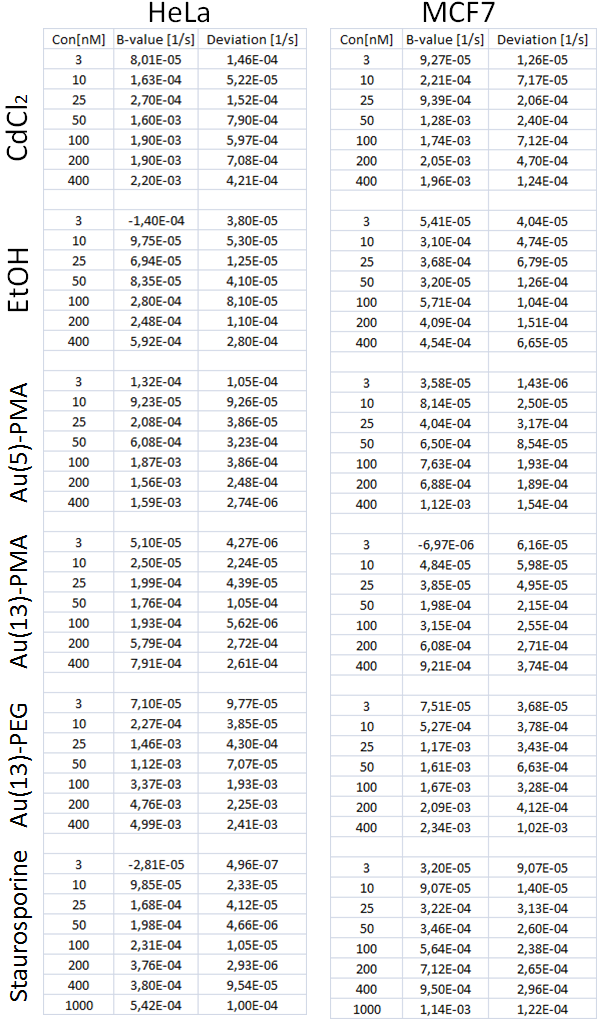


*Table S1: Mean B-values as determined from double measurements for each agent and the respective standard deviations.*

VIII. Raw data of pure medium and PBS

After each measurement, PBS was used for rinsing the chamber and the cantilever, and then the chamber was filled with cell medium, so that next measurement could be done. During this time the real-time deflection demonstrates current state of the cantilever and chamber for checking and re-calculating the equilibrium. In order to indentify the reference of initial position the measurements with pure buffer were recorded during 4 hours.







*Figure S10: Raw data of deflection vs. time with pure medium and PBS. The diagram (left) indicates the deflection with pure cell-medium during 4 hours. The diagram (right) indicates the deflection with pure PBS during 4 hours.*

Based on the deflection of equilibrium with same time scale cell-attachment and cell-detachment can be real-time monitored and clearly recorded for further analysis.

IX) Reference assay

*Figure S11: Cell viability of HeLa cells exposed to Au NPs. Cell viability was assessed by the resazurin assay upon exposure of HeLa cells to Au(5)-PMA, Au(13)-PMA, and Au(13)-PEG for 4 h, with CO_2_ (A, C, E), and without CO_2_ (B, D, F). Results are presented as mean cell viability ± SD from three independent experiments.*


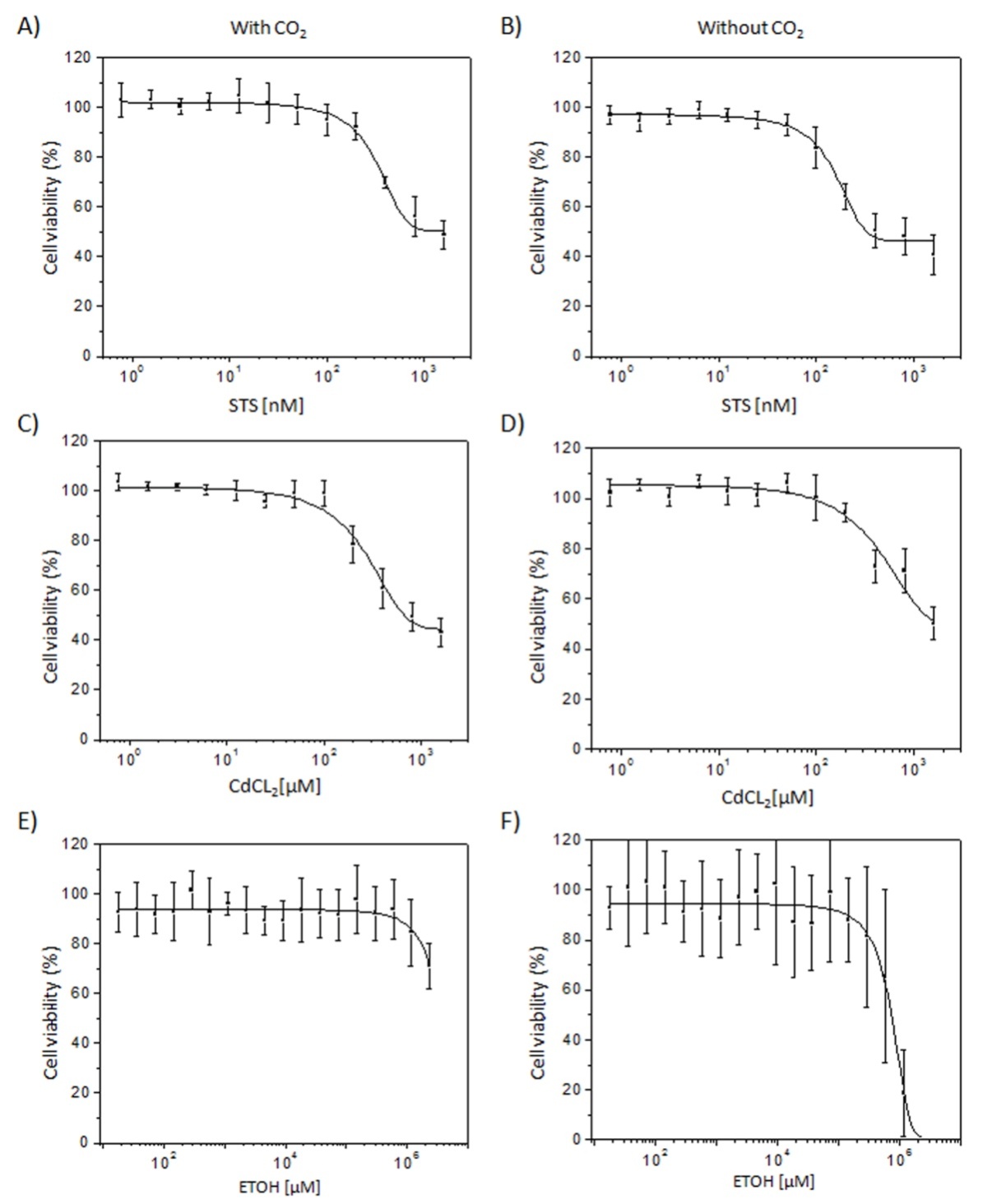


*Figure S12: Cell viability of HeLa cells exposed to chemical agents. Cell viability was assessed by the resazurin assay upon exposure of HeLa cells to STS (Staurosporine), EtOH, and CdCl_2_ for 4 h with CO_2_ (A, C, E), and without CO_2_ (B, D, F). Results are presented as mean cell viability ± SD from three independent experiments.*


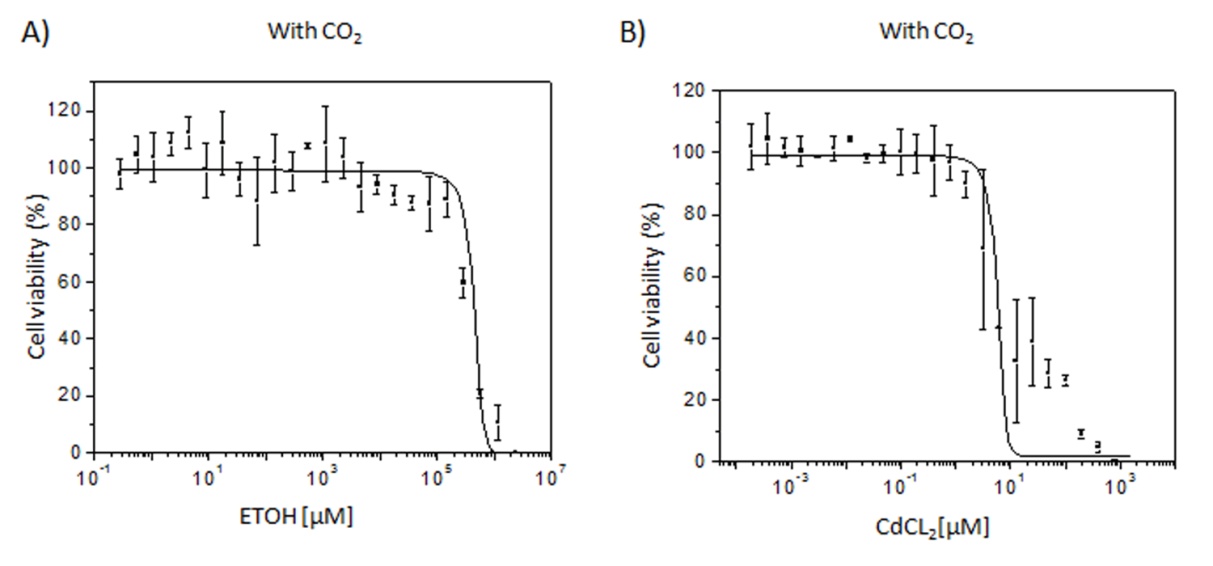


*Figure S13: Cell viability of HeLa cells exposed to chemical agents. Cell viability was assessed by the resazurin assay upon exposure of HeLa cells to EtOH and CdCl_2_ for 24 h with CO_2_ (A, B). Results are presented as mean cell viability ± SD from three independent experiments.*

*Figure S14: Cell viability of MCF7 cells exposed to Au NPs. Cell viability was assessed by the resazurin assay upon exposure of MCF7 cells to Au(5)-PMA, Au(13)-PMA, and Au(13)-PEG for 4 h, with CO_2_ (A, C, E), and without CO_2_ (B, D, F.) Results are presented as mean cell viability ± SD from three independent experiments.*

*
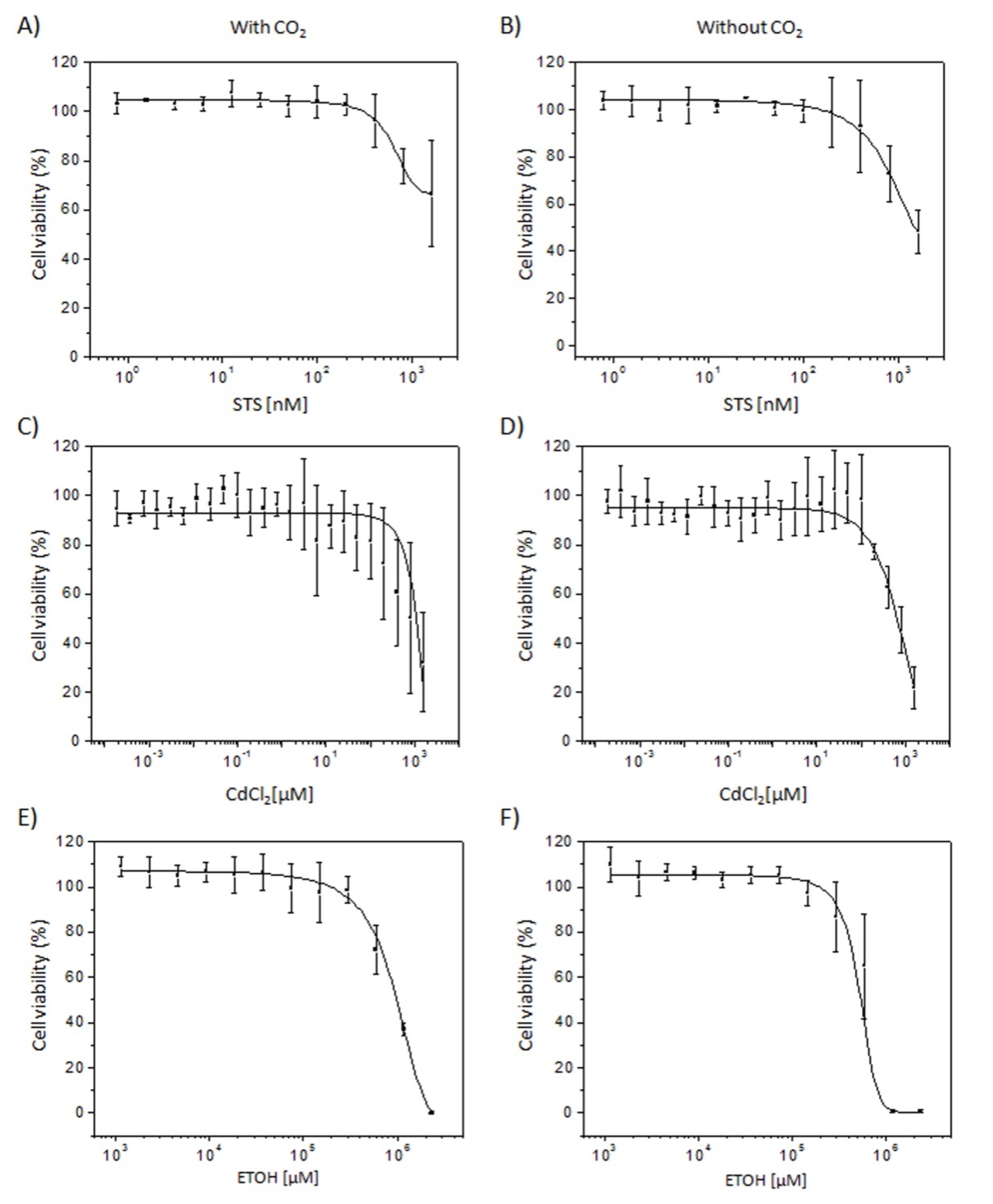
*

*Figure S15: Cell viability of MCF7 cells exposed to different chemical agents. Cell viability was assessed by the resazurin assay upon exposure of MCF7 cells to STS (Staurosporine), EtOH and CdCl2 for 4 h with CO_2_ (A, C, E), and without CO2 (B, D, F). Results are presented as mean cell viability ± SD from three independent experiments.*

*
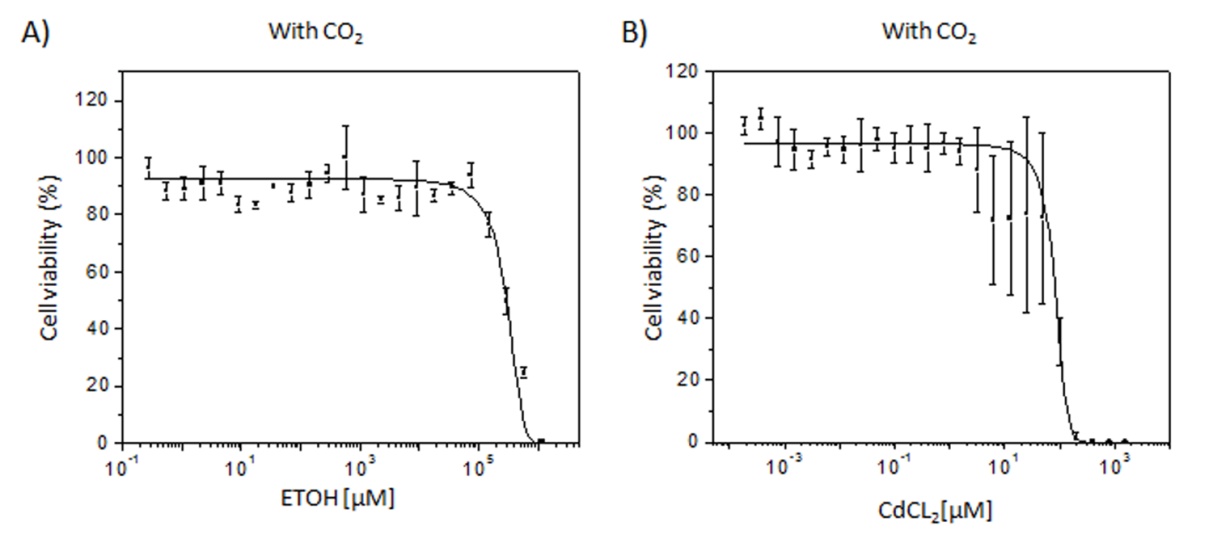
*

*Figure S16: Cell viability of MCF7 cells exposed to chemical agents. Cell viability was assessed by the resazurin assay upon exposure of MCF7 cells to EtOH and CdCl_2_ for 24 h with CO_2_ (A, B). Results are presented as mean cell viability ± SD from three independent experiments.*

X) Safety precautions when working with Staurosporine

Staurosporine is protein kinase inhibitor and toxic. CAS number: 62996-74-1, Alfa Aesar.

XI) References

1. Longo G, Alonso-Sarduy L, Rio LM, Bizzini A, Trampuz A, Notz J, Dietler G, Kasas S. Rapid detection of bacterial resistance to antibiotics using AFM cantilevers as nanomechanical sensors. Nat Nanotechnol. 2013; 8: 522-526.

2. A.Budó. Theoretische Mechanik: 11 edn. Berlin: VEB Deutscher Verlag der Wissenschaften; 1987.
